# Epidemiology and Risk Factors for Invasive Fungal Infections in Pancreas Transplant in the Absence of Postoperative Antifungal Prophylaxis

**DOI:** 10.1093/ofid/ofad478

**Published:** 2023-09-26

**Authors:** Jessica Zachary, Jeanne M Chen, Asif Sharfuddin, Muhammad Yaqub, Andrew Lutz, John Powelson, Jonathan A Fridell, Nicolas Barros

**Affiliations:** Department of Pharmacy, Indiana University Health, Indianapolis, Indiana, USA; Department of Pharmacy, Indiana University Health, Indianapolis, Indiana, USA; Department of Medicine, Indiana University School of Medicine, Indianapolis, Indiana, USA; Department of Medicine, Indiana University School of Medicine, Indianapolis, Indiana, USA; Department of Surgery, Indiana University School of Medicine, Indianapolis, Indiana, USA; Department of Surgery, Indiana University School of Medicine, Indianapolis, Indiana, USA; Department of Surgery, Indiana University School of Medicine, Indianapolis, Indiana, USA; Department of Medicine, Indiana University School of Medicine, Indianapolis, Indiana, USA

**Keywords:** epidemiology, fungal prophylaxis, invasive fungal infection, pancreas transplant, risk factors

## Abstract

**Background:**

Invasive fungal infections (IFIs) remain a rare yet dreaded complication following pancreas transplantation. Current guidelines recommend antifungal prophylaxis in patients with 1 or more risk factors. At our center, single-dose antifungal prophylaxis is administered in the operating room but none subsequently, regardless of risk factors. Here we evaluate the 1-year incidence, outcome, and risk factors associated with IFI following pancreas transplantation.

**Methods:**

A retrospective, single-center cohort study was conducted in patients who underwent pancreas transplantation between 1 January 2009 and 31 December 2019. Records were manually reviewed, and cases were adjudicated using consensus definitions. The 1-year cumulative incidence, mortality, and risk factors were analyzed by Kaplan-Meier method and differences between populations were assessed with Fisher test and Mann-Whitney *U* test.

**Results:**

Three hundred sixty-nine recipients were included. Twelve IFIs were identified: candidiasis (8), aspergillosis (2), histoplasmosis (1), and cryptococcosis (1). Intra-abdominal infections were the most common presentation (5), followed by bloodstream infections (3), disseminated disease (2), pulmonary disease (1), and invasive fungal sinusitis (1). Median time to IFI was 64 days (interquartile range, 30–234 days). One-year cumulative incidence was 3.25% (95% confidence interval, 1.86%–5.65%). There were no significant differences between patients with or without IFI regarding type of transplant (*P* = .17), posttransplant dialysis (*P* = .3), rejection (*P* = .5), cytomegalovirus serostatus (*P* = .45), or reoperation (*P* = .19). For patients with IFI, the 1-year graft and patient survival rates were 58% versus 95% (*P* < .0001) and 75% versus 98.6% (*P* < .001), respectively.

**Conclusions:**

Our study suggests that the use of a single-dose antifungal prophylaxis administered in the operating room but none subsequently does not result in an increased incidence of IFI following pancreas transplantation.

## Graphical Abstract

**Graphical abstract:**
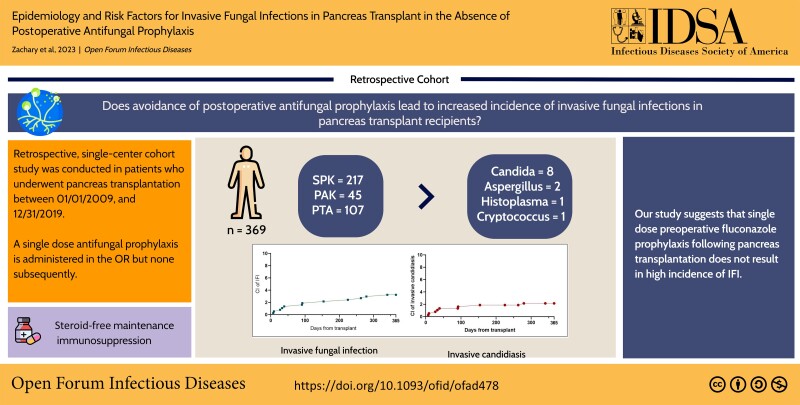
This graphical abstract is also available at Tidbit: https://tidbitapp.io/tidbits/epidemiology-and-risk-factors-for-invasive-fungal-infections-in-pancreas-transplant-in-the-absence-of-post-operative-antifungal-prophylaxis-f903a48a-e49a-42bd-a33b-49b3c7312c83

## Teaser

Invasive fungal infections (IFIs) remain a dreaded complication following pancreas transplantation. At our center, single-dose antifungal prophylaxis is administered in the operating room but none subsequently. This prophylactic strategy does not result in an increased incidence of IFI.

Pancreas transplantation is an established treatment for select patients with insulin-dependent diabetes [1–3]. While short- and long-term outcomes have steadily improved over the past decades, infectious complications continue to be common during the first year following transplantation [4–9]. Among those, invasive fungal infections (IFIs) remain a dreaded complication leading to increased morbidity and mortality [10].

Current guidelines from the Infectious Diseases Community of Practice of the American Society of Transplantation (IDCOP/AST) recommends antifungal prophylaxis in patients with enteric drainage, postperfusion pancreatitis, and vascular thrombosis [11]. However, the use of antifungal medications, particularly the use of azoles, are not without risk as they can cause significant hepatotoxicity, may lead to important drug–drug interactions (particularly with calcineurin inhibitors), and can facilitate the emergence of azole-resistant *Candida* spp [12].

Maintenance prednisone therapy is associated with an increased risk of fungal infections [13]. Since 2002, a maintenance immunosuppression regimen including a rapid corticosteroid wean has been successfully used in pancreas transplant recipients at Indiana University [14, 15]. Since then, fungal prophylaxis consists of a single dose of fluconazole administered in the operating room but none subsequently, regardless of risk factors. Here we describe the incidence, type, and impact of IFIs in the first year following pancreas transplantation in recipients who received single-dose fluconazole prophylaxis.

## METHODS

### Participants and Study Design

We retrospectively assessed a cohort of adult patients (≥18 years old) who underwent either simultaneous pancreas kidney transplant (SPK), pancreas after kidney transplant (PAK), or pancreas transplant alone (PTA) at Indiana University between 1 January 2009 and 31 December 2019. The study was approved by the Indiana University Institutional Review Board.

### Data Collection and Definitions

This was a retrospective single-center study. All data were gathered from the inpatient electronic medical record (Cerner Powerchart), outpatient electronic record (Organ Transplant Tracking Record), and CareWeb (Indiana health integrated electronic records across multiple medical systems).

Records were manually reviewed. In addition, to ensure the complete collection of all episodes of IFI, we obtained electronic reports from Indiana University Health, which included all positive cultures up to 365 days posttransplant, all positive fungal serologies up to 365 days posttransplant (ie, *Histoplasma* antigen, *Blastomyces* antigen, *Coccidioides* antigen, *Coccidioides* antibody, *Cryptococcus* antigen, *Aspergillus* galactomannan antigen, and 1–3-β-d-glucan), any prescription of antifungals for any patient after postoperative day 1. To ensure that we identified all episodes of clinically significant rejection, we obtained electronic reports of all pathology reports and reviewed any patient who received methylprednisolone at a dose >125 mg daily or thymoglobulin from day 7 to day 365 after transplantation.

Baseline information including demographics, type of transplant, indication for transplant, and date of transplant were collected for all patients. IFI episodes were adjudicated using the European Organization for Research and Treatment of Cancer and the Mycoses Study Group Education and Research Consortium (EORTC/MSGERC) criteria [16]. Only the first proven or probable IFI episodes were included in the analysis. The source of the infection was adjudicated using Centers for Disease Control and Prevention and National Healthcare Safety Network Criteria [17]. In patients with a proven or probable IFI, treatment of the infection was also recorded. Additional information recorded included allograft rejection and treatment used for rejection, date and reason of graft failure, and cause of death. For assessment of risk factors, only the events occurring prior to the censoring event (IFI, death, or 365 days) were included.

Kidney graft loss was defined as requiring reinitiating renal replacement therapy. Pancreas graft loss was defined as requiring insulin repletion on a regular basis.

The primary endpoint of this study was to assess the incidence of IFI in the first year posttransplant in recipients who received a single-dose preoperative fluconazole prophylaxis.

Secondary endpoints include determining the rate of acute rejection rate at 1 year posttransplant, graft survival at 1 year posttransplant, patient survival rate at 1 year posttransplant, and risk factors associated with the development of IFIs.

### Pancreas Transplant Operation and Immunosuppression

All allografts were procured and prepared on the backbench using standard techniques [18, 19]. Of specific note, no donor bowel prep or duodenum irrigation was used. All pancreas allografts were implanted with systemic venous and enteric exocrine drainage and recipients received rabbit antithymocyte globulin (rATG) induction and steroid-free maintenance immunosuppression. Per institution protocol, all patients received rATG 1 mg/kg with methylprednisolone 125 mg premedication intraoperatively followed by rATG 1 mg/kg on postoperative day (POD) 1–4, with methylprednisolone premedication 125 mg on POD 1 and prednisone 20 mg on POD 2–4. Maintenance immunosuppression consisted of tacrolimus and sirolimus with target trough levels of 6–8 ng/mL and 3–5 ng/mL, respectively. All PTA recipients also received a single dose of rituximab 150 mg/m^2^ and were maintained on a triple drug regimen of tacrolimus, sirolimus, and mycophenolate mofetil 500 mg or mycophenolic acid 360 mg twice daily [14].

### Perioperative Antimicrobial Prophylaxis

Perioperative antimicrobial prophylaxis consisted of 1 dose of fluconazole 400 mg and ampicillin/sulbactam dosed on body weight. Aztreonam was used in place of ampicillin/sulbactam for penicillin-allergic patients. Postoperatively, PTA recipients received valganciclovir 900 mg daily (adjusted for renal function) while SPK and PAK recipients received valganciclovir 450 mg daily (adjusted for renal function) for cytomegalovirus (CMV) prophylaxis. Recipients who were CMV donor negative/recipient negative received acyclovir 400 mg twice daily instead of valganciclovir. All patients received sulfamethoxazole/trimethoprim single-strength tablet for *Pneumocystis jirovecii* prophylaxis. Patients with a sulfa allergy received either atovaquone or pentamidine inhalation in lieu of sulfamethoxazole/trimethoprim. All patients received deep venous thrombosis prophylaxis starting on POD 1 with lower extremity sequential compression devices until mobilizing. Systemic anticoagulation was reserved for patients with documented thrombophilia.

### Statistical Analysis

Descriptive statistics were used to evaluate baseline characteristics. The 1-year cumulative incidence, mortality, and risk factors were analyzed by Kaplan-Meier method and compared by the log-rank test, and differences between patient populations were assessed with Fisher test. Continuous variables were analyzed using Mann-Whitney *U* test. We performed the analysis including all patients with IFIs and also a second analysis in which we analyze the same variables but only in patients with invasive candidiasis (IC).

A 2-sided value of *P* ≤ .05 was considered significant. All statistical analyses were performed using GraphPad Prism version 9.3.1 (GraphPad Software, San Diego, California).

## RESULTS

### Baseline Characteristics

**Figure 1.**
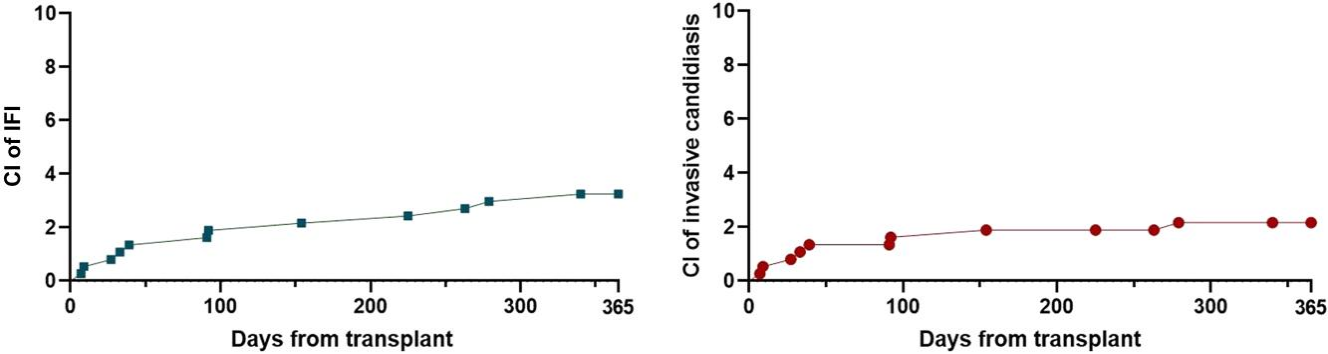
Cumulative incidence of all invasive fungal infections (left) and invasive candidiasis (right). In both panels, the y axis goes up to 10% to allow better visualization. Abbreviations: CI, cumulative incidence; IFI, invasive fungal infection.

A total of 369 patients underwent either SPK, PAK, or PTA between 1 January 2009 and 31 December 2019 at Indiana University Health, University Hospital and were included in the analysis. Demographic data for the study population are contained in Table 1 (IFI) and Table 2 (IC). The overall population was primarily male (53.4%) and White (91%). The mean age of the population was 44.2 years; 58.8% of patients received SPK, 29% received PTA, and 12.2% received PAK transplant. There were no differences in demographic characteristics between patients with or without IFI. Within the first year following transplant, 12 (3.25%) patients developed an IFI. The calculated cumulative incidence of IFI and IC at 1 year posttransplant was 3.25% (95% confidence interval [CI], 1.86%–5.65%) and 2.17% (95% CI, 1.09%–4.29%), respectively (Figure 1). The median time to IFI was 91 days. Invasive candidiasis occurred earlier with a median time of 36 days. Three IFI occurred in the first 30 days following transplant; all of those were due to *Candida glabrata*. IFI occurred in 6 PTA and 6 SPK. Of the IFIs, 10 were proven and 2 probable infections (Table 3). There were no differences in demographic characteristics between patients with or without IC. There were no significant differences between patients with or without IFI regarding type of transplant (*P* = .17).

**Table 1.** Demographics of Patients With Invasive Fungal Infections

Characteristic	All (N = 369)	IFI (n = 12)	No IFI (n = 357)	*P* Value
Age, y, mean ± SD	44.2 ± 10.3	47.9 ± 12.36	44.9 ± 10.19	.28
Race				.7
White	336 (91)	12 (100)	324 (90.7)	
African American	31 (8.4)	0 (0)	31 (8.7)	
Asian	1 (0.3)	0 (0)	1 (0.3)	
Unknown	1 (0.3)	0 (0)	1 (0.3)	
Sex (male)	197 (53.4)	5 (41.7)	192 (53.8)	.6
Type of transplant				.2
SPK	217 (58.8)	6 (50.0)	211 (59.1)	
PAK	45 (12.2)	0 (0.0)	45 (12.6)	
PTA	107 (29.0)	6 (50.0)	101 (28.3)	
CMV serostatus^a^				.4
High	183 (40.4)	4 (33.3)	179 (50.1)	
Moderate	35 (9.5)	1 (8.3)	34 (9.5)	
Low	151 (40.9)	7 (58.3)	144 (40.3)	
Posttransplant dialysis	11 (2.98)	1 (8.3)	10 (2.8)	.3
Rejection^b^	20 (5.4)	1 (8.3)	19 (5.3)	.5
Graft loss^c^ (death-censored)	13 (3.5)	2 (16.6)	17 (4.8)	.35
Cold ischemia time, min, mean ± SD	470 ± 233	495 ± 137	469 ± 236	.3
Operation time, min, mean ± SD	271 ± 106	301 ± 151	270 ± 104	.6
Reoperation	98 (27)	9 (75)	89 (25)	.19

Data are presented as No. (%) unless otherwise indicated.

Abbreviations: CMV, cytomegalovirus; IFI, invasive fungal infection; PAK, pancreas after kidney transplant; PTA, pancreas transplant alone; SD, standard deviation; SPK, simultaneous pancreas transplant.

^a^CMV serostatus: High represents CMV donor positive/recipient negative; moderate represents CMV recipient positive (regardless of donor CMV serostatus); low represents CMV donor negative/recipient negative.

^b^Only episodes of rejection prior to censoring event were included (ie, IFI, death, or 365 d).

^c^Graft loss includes all grafts lost at the end of follow-up (365 d or death).

**Table 2.** Demographics of Patients With Invasive Candidiasis

Characteristic	All (N = 369)	IC (n = 8)	No IC (n = 361)	*P* Value
Age, y, mean ± SD	44.2 ± 10.3	45.8 ± 13.58	44.9 ± 10.25	.7
Race				.7
White	336 (91)	8 (100)	328 (90.9)	
African American	31 (8.4)	0 (0)	31 (8.6)	
Asian	1 (0.3)	0 (0)	1 (0.25)	
Unknown	1 (0.3)	0 (0)	1 (0.25)	
Sex (male)	197 (53.4)	2 (25)	195 (54)	.15
Type of transplant				.54
SPK	217 (58.8)	3 (37.5)	214 (59.3)	
PAK	45 (12.2)	0 (0.0)	45 (12.4)	
PTA	107 (29.0)	5 (62.5)	102 (28.3)	
CMV serostatus^a^				.4
High	183 (40.4)	3 (37.5)	180 (49.9)	
Moderate	35 (9.5)	0 (0.0)	35 (9.7)	
Low	151 (40.9)	5 (62.5)	146 (40.4)	
Posttransplant dialysis	11 (2.98)	0 (0.0)	11 (3.0)	*>*.9
Rejection^b^	20 (5.4)	1 (12.5)	19 (5.3)	.36
Graft loss^c^ (death-censored)	13 (3.5)	2 (25)	11 (4.7)	.06
Cold ischemia time, min, mean ± SD	470 ± 233	476 ± 96	468 ± 235	.3
Operation time, min, mean ± SD	271 ± 106	349 ± 163	271 ± 106	.11
Reoperation	98 (27)	4 (50)	93 (25)	.19

Data are presented as No. (%) unless otherwise indicated.

Abbreviations: CMV, cytomegalovirus; IC, invasive candidiasis; PAK, pancreas after kidney transplant; PTA, pancreas transplant alone; SPK, simultaneous pancreas transplant.

^a^CMV serostatus: High represents CMV donor positive/recipient negative; moderate represents CMV recipient positive (regardless of donor CMV serostatus); low represents CMV donor negative/recipient negative.

^b^Only episodes of rejection prior to censoring event were included (ie, invasive fungal infection, death, or 365 d).

^c^Graft loss includes all grafts lost at the end of follow-up (365 d or death).

**Table 3.** Treatment of Invasive Fungal Infections

Type of Transplant	Class of IFI	Site of Infection	Organism	Days Since Transplant	Diagnostic Criteria	Treatment and Management	Outcome
SPK	Proven	Blood	*Candida glabrata*	9	Positive blood culture	Micafungin followed by fluconazole	No effect on allograft
SPK	Proven	Blood	*Candida glabrata*	92	Positive blood culture	Micafungin	Patient death with functioning graft (post-Tx: 208 d)
SPK	Proven	Blood	*Candida glabrata*	33	Positive blood culture	Micafungin	No effect on allograft
PTA	Proven	Blood	*Cryptococcus neoformans*	225	Positive blood culture and serum cryptococcal antigen >1:2560	Liposomal amphotericin B + flucytosine	Patient death with functioning graft (post-Tx: 242 d)
SPK	Proven	Abdominal abscess	*Candida glabrata* ^a^	27	Positive culture	Micafungin	No effect on allograft
PTA	Proven	Deep surgical site	*Candida glabrata* ^b^	7	Positive culture	Micafungin followed by fluconazole	Allograft pancreatectomy due to thrombosis and decreased functionality over the course of hospital
PTA	Proven	Tissue culture	*Candida albicans*	279	Positive culture	Surgical debridement	No effect on allograft
PTA	Proven	Abdominal abscess	*Candida albicans*	154	Positive culture	Percutaneous drainage	No effect on allograft
SPK	Proven	Peritoneal fluid	*Candida albicans*, *Candida glabrata*^c^	39	Positive culture	Fluconazole	No effect on allograft
PTA	Proven	Invasive fungal sinusitis	*Aspergillus* spp	340	Positive culture + biopsy	Voriconazole	Patient death with functioning graft (post-Tx: 357 d)
SPK	Probable	Pulmonary	*Aspergillus* spp	263	BAL galactomannan 1.1 ng/mL + pulmonary nodule	Voriconazole	No effect on allograft
PTA	Probable	Disseminated	*Histoplasma* spp	91	Urine histoplasma antigen: 10.88 ng/mL	Itraconazole	No effect on allograft

Abbreviations: IFI, invasive fungal infection; PTA, pancreas transplant alone; SPK, simultaneous pancreas transplant.

^a^Cultures isolated *Candida glabrata* and *Enterococcus faecium*.

^b^Cultures isolated *Candida glabrata*, *Klebsiella oxytoca*, and *Streptococcus viridans* group.

^c^Cultures isolated *Candida glabrata* and *Enterococcus faecium*.

### Clinical Presentation, Microbiology, and Outcomes of IFI

Invasive candidiasis was the most common IFI (8/12). A single organism was responsible for 7 of these infections; cultures from 1 recipient grew >1 *Candida* spp. *Candida glabrata* (n = 6) and *Candida albicans* (n = 3) were isolated in cultures. Other IFIs included histoplasmosis (1/12) and aspergillosis (2/12). Intra-abdominal infections were the most common presentation (5/12), followed by bloodstream infections (3/12), disseminated disease (2/14), pulmonary disease (1/12), and invasive fungal sinusitis (1/12). The treatment of each IFI is described in Table 2.

Two patients were managed with surgical debridement without any systemic antifungal therapy. One of those patients had a small peripancreatic abscess, which was completely drained during surgery. Cultures isolated *Candida albicans*. The post-op was uneventful and antifungal therapy was not started.

The other patient underwent emergent laparotomy for small bowel obstruction secondary to perforated appendix. An abscess was identified during the surgery and drained completely, and a Jackson-Pratt drain was placed. The drain was removed within the first week following the surgery.

The 1-year cumulative pancreas allograft survival rate (death-censored) was 58% (95% CI, 27%–80%) in patients who experienced an IFI versus 95% (95% CI, 92%–97%) in patients who did not (*P* < .0001). All episodes of rejection occurred prior to the censoring event (ie, IFI, death, or 365 days). Hence, all episodes of rejection were included in the analysis for risk factors. Allograft rejection was not associated with IFI (*P* = .5). The overall rate of rejection at 1 year was 5.5% for all patients (IFI: 8.3% vs non-IFI: 5.3%; *P* = .36). All episodes of rejection were biopsy proven and included either graft (ie, kidney or pancreas). Among the 20 episodes of rejection, 18 were biopsy-proven kidney rejection, 1 was biopsy-proven pancreas rejection, and 1 was biopsy-proven kidney rejection with presumed pancreas rejection.

Three patients with IFI died during the study period. One recipient died after a prolonged hospital stay, which was complicated by enterocutaneous fistula, gastrointestinal bleed from anastomosis site, liver injury, acute kidney injury requiring hemodialysis, respiratory failure due to multiple bacterial pneumonias with multidrug-resistant organisms (carbapenem-resistant *Pseudomonas aeruginosa* and *Enterobacter cloacae* complex), and disseminated cryptococcal infection. The patient developed disseminated cryptococcal infection 225 days posttransplant and despite 14 days of flucytosine and liposomal amphotericin treatment, his overall clinical status continued to decline and care was withdrawn. The second recipient death was due to an invasive fungal sinusitis due to *Aspergillus* spp that occurred 340 days posttransplant and failed treatment with voriconazole, liposomal amphotericin B, and micafungin. The third recipient death occurred due to exsanguination in the setting of common iliac artery fistula into the sigmoid colon. The patient died 208 days after the transplant.

The 1-year patient survival rate was significantly lower in patients with IFI compared with patients who did not develop an IFI (75% vs 98.6%, respectively; *P* < .002).

### Risk Factors for IFI

Additional known risk factors for the development of IFI and poor graft outcomes were also examined including CMV serostatus, posttransplant renal replacement therapy, cold ischemia time, operation time, reoperation, and rejection (Table 1). Reoperation and rejection were included as risk factors only if they occurred prior to the censoring event. Reoperation occurred commonly in patients with IFI compared to non-IFI patients (42% vs 25%, respectively) though it was not statistically significant (*P* = .19). Rejection occurred in 8.3% of IFI and in only 5.3% of non-IFI patients (*P* = .49). No other significant differences were found between patients with or without IFI (Table 1).

Given that the pathophysiology and risk factors for different fungal infections may differ, we also analyzed the IC separately. Reoperation occurred in 50% of patients with IC versus 21% of patients without IC (*P* = .21). Rejection occurred in 12.5% of patients with IC versus 5.3% of patients without IC (Table 2).

## DISCUSSION

In this study, we examined the 1-year cumulative incidence of IFI in pancreas transplant recipients who received a single dose of fluconazole administered in the operating room but none subsequently, regardless of risk factors.

The results of this retrospective, single-center, cohort study suggest that avoidance of antifungal prophylaxis beyond the operating room for pancreas transplantation resulted in a similar 1-year incidence of IFI to prior reports. We did not identify any risk factors that were associated with IFI.

In a cohort of 15 centers in the United States from 2001 to 2006 investigating 1213 pancreas transplant recipients, the 1-year cumulative incidence of IFI was estimated to be 4.0% [10]. Subsequently, a large Swiss cohort of 3541 solid organ transplant recipients described that 7.4% of the patients developed an IFI during the first year. However, this cohort only included 73 pancreas transplant recipients, of which 13.7% developed an IFI [7]. Neither report described their antifungal prophylaxis regimen.

In 1996, Benedetti et al retrospectively studied 445 consecutive pancreatic transplantations and found that intra-abdominal fungal infections occurred in 9.2% of all cases. In addition, they showed that patients with enteric drainage had 2 times higher rates of fungal infections compared to bladder-drained transplantations. Other identified risk factors included vascular thrombosis and postperfusion pancreatitis. Furthermore, the rate of fungal infections was only 6% if fluconazole prophylaxis was given compared to 10% in those without prophylaxis. Based on these results, the IDCOP/AST recommends systemic antifungal prophylaxis for enteric-drained pancreas transplant recipients (which, according to the International Pancreas Transplant Registry, represents the majority [>95%] of pancreas transplants performed currently) and those complicated with vascular thrombosis or postperfusion pancreatitis [11, 20]. However, significant advances in surgical techniques and immunosuppressive regimens have occurred since then [20].

In a recent small single-center, retrospective cohort study, Shaikh et al showed a 1-year cumulative incidence of 3.6% despite the lack of any systemic antifungal prophylaxis. They reported 2 cases of IFI at 1 year, among 56 pancreas transplant recipients [21]. However, the limited number of events renders extrapolation to other cohorts difficult. In our study, we followed a total of 369 patients for 1 year posttransplant and observed IFI in 12 (3.25%) patients.

In our large cohort, despite the lack of postoperative antifungal prophylaxis, we reported similar or lower incidence of IFIs to other reports [7, 10, 21]. The makeup of IFIs found in our patient population is similar to what is reported in literature where *Candida* spp are the most common cause of IFI [11]. In prior reports, *Candida* spp account for 49%–85% of all IFIs [11]. In our study population, 66.6% of IFIs were caused by *Candida* spp. Unlike prior reports where *Candida albicans* is the most common isolated *Candida* sp, *Candida glabrata* represented the most common *Candida* sp in our cohort [11, 22]. It is uncertain if the shift is secondary to the use of fluconazole in the perioperative period or associated to shifting epidemiology in our center.

Our study does not support the recommendation from the IDCOP/AST as no patient received antifungal prophylaxis following transplantation regardless of risk factors and the observed incidence of IFI was similar or lower to that reported literature [11]. While the mortality in patients with IFI was higher than that of patients without IFI, only 1 death was directly attributable to the IFI. Furthermore, all deaths occurred >200 days posttransplantation. For these reasons, it is unlikely that the use of postoperative antifungal prophylaxis would have had any impact on these cases. The only death occurring in patients with IC was not attributable to the infection as it was related to exsanguination in the setting of common iliac artery fistula into the sigmoid colon. This event occurred >100 days following the IC.

Two patients with IC had graft failure. One was related to a graft arterial thrombosis and the second was related to splenic artery thrombosis with pancreatic tail necrosis and a polymicrobial peripancreatic infection. While in both cases the fungal infections may have contributed to the graft loss, it is unclear if they were the main factor leading the process and whether prophylaxis would have prevented the graft loss. The risk factors and pathophysiology of IC may differ from those of other IFIs, including endemic mycoses and molds (eg, *Aspergillus* spp). We assessed risk factors for the development of IC by performing a subgroup analysis. We did not identify any risk factors that were associated with IC. We did not include CMV disease as a risk factor as none of our patients with IC had CMV disease prior to the infection.

Two patients did not receive systemic antifungal therapy despite having proven IFIs. This raises the question of whether these patients were appropriately classified. Given the retrospective nature of the study, we are unable to identify any further data that would allow us to further delineate the classification of the patients. For that reason, we decided to err on the side of including them as part of our study population. If we exclude those patients, our conclusion may be stronger as the cumulative incidence of invasive infections without those patients would be even lower.

Our study does carry significant limitations that are not only restricted to its retrospective design. The low number of events identified (IFIs) prohibits the use of multivariate analysis and makes it difficult to detect differences between those with and without IFI, putting it at risk for type II error. Furthermore, this was a single center and results may not be applicable to other centers as immunosuppression regimens, surgical techniques, and antifungal prophylaxis practices may differ.

## CONCLUSIONS

Our study suggests that the avoidance of postoperative fungal prophylaxis following pancreas transplantation does not result in a high incidence of IFIs. Furthermore, we did not identify any risk factor that is associated with the development of an IFI. The use of a single dose of fluconazole prophylaxis in the operating room but none subsequently is advantageous because it eliminates drug–drug interactions, particularly with “azoles” and immunosuppression medications such as tacrolimus, cyclosporine, sirolimus, and everolimus, and decreases the emergence of azole-resistant *Candida* spp. Since our center does not use steroids as part of maintenance immunosuppression regimens, these results may not be applicable to centers with immunosuppression regimens that include steroids. Additional larger multicenter studies are warranted to determine whether these data are generalizable to other transplant centers with different immunosuppression regimens.

## Notes

***Patient consent.*** Given the retrospective nature of this work, the Indiana University Institutional Review Board (IRB) approved a waiver of consent for the conduct of this study. The design of the work has been approved by the Indiana University IRB and conforms to standards currently applied in the United States.
